# Oxychloride of Zinc

**Published:** 1897-11

**Authors:** S. T. Kirk

**Affiliations:** Kokomo, Ind.


ARTICLE IV.

### OXYCHLORIDE OF ZINC.

BY S. T. KIRK, D. D. S, KOKOMO, IND.

This, the oldest of our cements and usually known as os artificial, is, in its proper use, the very best of the cement family. There are preparations of the oxyphosphates that will stand in exposed places better, but there are none which have so good preserving qualities.

In the days of long ago, more than a quarter of a century, the older members of the profession remember it as the only cement at our command. It is from the observance of its use in the past that I wish to speak. Having practiced in the same place for more than thirty years, I have had a good chance for observation.

Early in my professional life I learned that many failures came from the filling of roots with gold, and even from the most eminent practitioners; circumstances led me to fill roots with os artificial instead. And in after years I have learned its true value, especially since crowning has been in such common use. I find many roots of teeth that were so filled, the crowns having been entirely broken down to the line where the os artificial protected it, and then remained in good condition, waiting to be redeemed and placed to good use in supporting the modern crown. I have found many roots buried by the gum, and, after consulting my record and finding they were filled with this material, I have immediately disposed of the gum and found the root solid and able to hold a useful crown. I use that preparation of oxychloride of zinc known as os artificial, and for many years I filled the entire root with it, but often, if great care is not used, the fluid will pass through the foramen and cause inflammation of the periosteum; hence, on the introduction of gutta-percha for root-filling, I fill the canals partially with it, but invariably use the os artificial for the main part of pulp-chamber. I think the fluid permeates the tubuli and preserves the whole tooth-structure as nothing else can. For the same reason I use it in all large cavities, covering the nearly exposed pulp with a non-irritant. I fill the cavity one-half or two thirds full of the os artificial, pressing it solidly into its place with spunk. This cannot be done with the oxyphosphates.

Thus far it makes a better filling to preserve the tooth-structure than any metal, and at the same time makes a solid and even foundation upon which to build the metal, which need be no more, in many cases, especially large crown cavities, than as a cork to a bottle.

In filling a root having an alveolar abscess with fistulous opening, if a small quantity of the fluid passes through the foramen and the canal is immediately filled with cement, it invariably becomes sound and well in a short time, and remains so.

The two points to be guarded against are: first, in root-filling, not to let the fluid pass through, as it will inflame the periosteum; second, to protect the nearly exposed pulp in large cavities.

A piece of cotton moistened with the fluid is most excellent to wipe out all cavities before filling, on account of its preserving qualities.—*American Dental Weekly.*

